# An Unusual Case of Pleuropulmonary Blastoma in a Child with Jejunal Hamartomas

**DOI:** 10.1155/2013/140508

**Published:** 2013-07-22

**Authors:** Chantal Lucia-Casadonte, Sakil Kulkarni, Ricardo Restrepo, Ruben Gonzalez-Vallina, Carole Brathwaite, Edward Y. Lee

**Affiliations:** ^1^Department of Medical Education, Miami Children's Hospital, Miami, FL 33155, USA; ^2^Department of Radiology, Miami Children's Hospital, Miami, FL 33155, USA; ^3^Department of Pediatric Gastroenterology, Miami Children's Hospital, Miami, FL 33155, USA; ^4^Department of Pediatric Pathology and Clinical Pathology, Miami Children's Hospital, Miami, FL 33155, USA; ^5^Department of Radiology, Children's Hospital Boston and Harvard Medical School, 300 Longwood Avenue, Boston, MA 02115, USA

## Abstract

We report a rare case of 9-month-old girl who presented with a choking episode and was found to have an incidental finding of a lung cyst and iron deficiency anemia leading to the diagnosis of pleuropulmonary blastoma (PPB) and a jejunal hamartoma. Our patient is the eighth that has been reported with the association of PPB with jejunal hamartoma and the first one in the radiological literature. PPB is the pulmonary analog of other dysontogenetic neoplasms in childhood. A biological sequence has been described with the three types of PPB to be interrelated as part of pathologic progression. PPB can be associated with other cysts and/or neoplasms in different organs. PPB is part of a hereditary neoplasia predisposition syndrome in up to 40% of cases. Mutations in DICER gene have been described with PPB. Hence, a pediatric patient diagnosed with PPB should be screened for associated conditions during childhood and adolescence including intestinal polyps. Obtaining family history for other neoplasms or cysts is important information that should raise the possibility of PPB in pediatric patients with cystic lung lesions. The presence of this syndrome should alert the clinician to screen and follow up patients and their relatives.

## 1. Case Report

**Figure 1:**
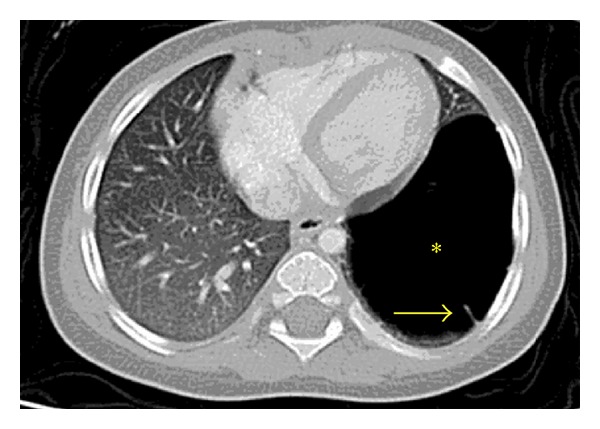
Axial lung window CT image shows a large thin walled cyst (asterisk) with a single septation (arrow) in the left lower lobe that is proved to be a type I PPB.

A 9-month-old Hispanic female with no significant past medical history presented to the emergency room after a choking episode, followed by a brief episode of hypotonia while playing in the park. Physical examination revealed only mild respiratory distress with decreased breath sounds in the left hemithorax. She was also found to have iron deficiency anemia, with hemoglobin of 6.9 g/dL, hematocrit of 22.9%, mean corpuscular volume of 68.3 fL, and iron level of 23 mcg/dL; stool guaiac test was positive. Meckel's scan was negative. On a chest radiograph, a large round lucency was seen in the left lower lobe measuring 4.2 cm × 5.3 cm. A computerized tomography (CT) angiogram of the chest was obtained confirming the presence of a large cystic lesion in the left lower lobe with a single septation and no solid component or abnormal vascular supply (Figure 1). In addition, several small cysts were seen in the ipsilateral upper lobe. 

**Figure 2:**
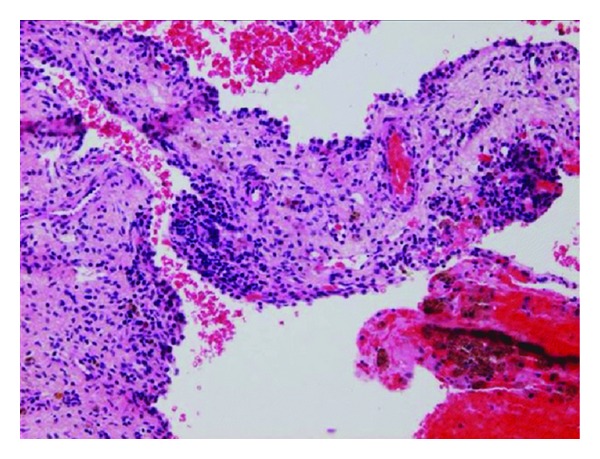
Surgical resection specimen demonstrates focal mesenchymal hypercellularity consistent with pleuropulmonary blastoma type 1 (H&E; original magnification ×100).

Patient underwent left lower lobe segmentectomy and a wedge resection of the left upper lobe. An abdominal ultrasound (US) was obtained for persistence of anemia which showed a small bowel intussusception with an approximately 2.5 × 1.8 × 1.5 cm soft-tissue lesion acting as a leading point (Figure 2). CT enterography was then obtained confirming the US findings. No other abnormalities were seen in both abdominal US and CT. Interestingly, the child did not have any intermittent irritability or abdominal distension.

**Figure 3:**
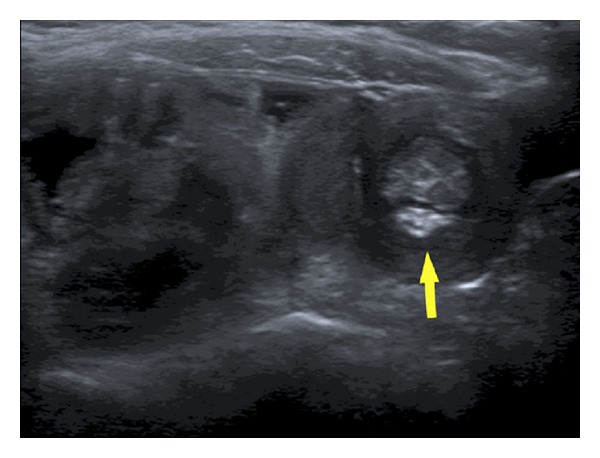
Transverse US image demonstrates a small bowel intussusceptions in the left lower quadrant containing an echogenic leading point (arrow) corresponding to the leading point.

The histopathological evaluations of the cystic lung lesions showed cysts separated by thin walled septa lined by ciliated columnar respiratory epithelium with subepithelial condensations of mesenchymal tumor cells giving a sarcoma botryoid-like appearance (Figure 3). These findings are consistent with Type 1 PPB. A jejunal polyp was resected with a pathologic diagnosis of a hamartomatous polyp. The patient is currently undergoing chemotherapy after the diagnosis of Type I PPB was confirmed.

## 2. Discussion

Pleuropulmonary blastoma (PPB) is a rare mesenchymal tumor, affecting primarily pediatric patients under 6 years of age [1]. A family history of a neoplasm or dysplasia in other organs is highly suggestive of this disease, in addition to spontaneous pneumothorax in a patient with a cystic pulmonary lesion. PPB can be genetically determined in some cases and is associated with a distinctive inherited cancer predisposition syndrome [2].

PPB is unique among pediatric cancers because of three main reasons. First, a biological sequence has been described with the three types of cyst-PPB to be interrelated as part of pathologic progression. Second, PPB can be associated with other cysts and/or neoplasms in different organs. And third, PPB is part of a hereditary neoplasia predisposition syndrome in up to 40% of cases [3, 4]. Our case not only fulfills the first two of these three intriguing characteristics but also had the infrequent association with intestinal polyps. 

Juvenile hamartomatous polyps can be an isolated entity or a part of a syndrome like Peutz Jeghers syndrome, Cowden syndrome, Juvenile Polyposis Syndrome, and so forth. The various gene defects associated with juvenile polyps are LKB1, PTEN, SMAD4, and so forth [5]. The atypical features of the juvenile polyps in our case were the age of onset (infancy) and intussusception at presentation. 

The association of intestinal polyps and PPB is rare and, so far, it has been reported in only seven children by data from the PPB registry by Priest et al. [6]. Five of the seven children had associated cystic nephroma (CN) along with PPB and multiple juvenile intestinal polyps. In the majority of these cases, the polyps were in the small bowel and most presented with intussusception. The data from the International Pleuropulmonary Blastoma Registry (IPPBR) indicates that 40% of the cases with PPB may have a genetic predisposition [3]. Recently, PPB has been linked to mutations in the *DICER-1* gene. This gene encodes an enzyme that negatively regulates gene expression [7]. To the best of the authors' knowledge, the association of PPB and juvenile hamartomatous polyps has not been described in the pediatric gastroenterology literature. 

The mainstay of therapy in PPB type 1, as seen in our case, is surgical excision followed by chemotherapy [6]. Once diagnosed with Type I PPB, the IPPBR recommends surveillance with chest radiographs monthly and chest CT every three months for the first two years after diagnosis or until child is 60 months of age [6]. Some investigators propose that chest CT should be extended to include the kidneys and bowel [3]. The overall survival of Type 1 PPB after 5 years is around 85%, while the recurrence free survival is around 70%. The overall and recurrence free survival are significantly lower for Type 2 and Type 3 PPB, compared to type 1 PPB.

A pediatric patient diagnosed with intestinal polyps should be screened for associated conditions during childhood and adolescence [6]. The main purpose of this case report is to raise the awareness of the association of jejunal hamartomas with a lethal condition like PPB. Family history for other neoplasms or cysts is important information which raises the possibility of PPB in pediatric patients with intestinal polyps and cystic lung lesions. The age of onset for screening, the frequency of screening, and the spectrum of PPB-associated conditions for which screening of the family members might be appropriate are still unclear [3, 6].
